# Influence of bioaugmentation on biodegradation of phenanthrene-contaminated soil by earthworm in lab scale

**DOI:** 10.1186/s40201-014-0150-2

**Published:** 2014-12-24

**Authors:** Hosseinali Asgharnia, Ahmad Jonidi Jafari, Roshanak Rezaei Kalantary, Simin Nasseri, Amirhossein Mahvi, Kamyar Yaghmaeian, Ali Esrafili, Yousef Dadban Shahamat

**Affiliations:** Department of Environmental Health Engineering, School of Public Health, Iran University of Medical Sciences, Tehran, Iran; Department of Environmental Health Engineering, School of Public Health, Tehran University of Medical Sciences, Tehran, Iran; Center for Water Quality Research, Institute of Environmental Research, Tehran University of Medical Sciences, Tehran, Iran; Department of Environmental Health Engineering, School of Public Health, Golestan University of Medical Sciences, Gorgan, Iran

**Keywords:** Bioaugmentation, Biodegradation, Phenanthrene, Earthworm and bacteria

## Abstract

**Background:**

Use of earthworm to eliminate the phenanthrene from the soil (bioaccumulation) is developed as an economical method. Bioaugmentation of microorganism was used for promotion of bioaccumulation by earthworm. The aim of this study was to determine the bioaccumulation or biodegradation of phenanthrene by *Eisenia fetida and* bacterial consortium in polluted soil.

**Methods:**

The amount of 0.4 kg of the polluted soil in the ratio of 10 and 30 mg phenanthrene per kg of dry soil was transferred into each pot. Afterwards, bacteria and earthworms were added to each pot in separate and combination. The samples were kept under field conditions, and the retention concentrations of phenanthrene were analyzed after 8 weeks.

**Results:**

Results showed that the *Eisenia fetida* was able to significantly remove phenanthrene from the polluted soil samples. Bioaccumulation and bioaugmentation alone have the removal efficiency of 60.24% and 50.3%, respectively. In the combined mode, phenanthrene removal efficiency was 63.81%.

**Conclusions:**

The current study indicated that the use of earthworms, could improve both phenanthrene bioavailability and microbial activity, which led to enhancing removal of carbon-based pollutants.

## Background

Polycyclic Aromatic Hydrocarbons (PAHs) are a group of more than 100 various chemicals substances formed during the incomplete burning of coal, oil, gas, rubbish and tobacco [1,2]. PAHs are known as the mutagen and carcinogen substance. They are toxic and persistent in the environment [3,4]. Methods based on the techniques used to purify the contaminated soils were divided into three categories: physical, chemical and biological [5,6]. Bioremediation based on the physical and chemical engineering technologies has high energy consumption and therefore their use is expensive, especially when a large volume of surface of soil is contaminated. The physical and chemical interactions of the soil structure are possibly damaged that is unusable for agriculture. A physical remediation method is led to eliminate the pollutants from soil - water composition, which can be used for further refining [7,8]. Advantage of chemical oxidation includes the use of on-site contamination. Due to the relatively low cost for degradation of pollution, the contamination is eliminated. Disadvantages of chemical oxidation are the ability of creating dangerous byproducts and added chemicals (such as hydrogen peroxide, manganese dioxide and iron oxide) which can have a negative impact on soil organisms or chemical composition. The advantages of bioremediation are the possibility of cleaning the site and the lack of relocation and transportation of toxic waste. Ensure that the permanent removal of polluting and combining of this technology to other methods of purification, the survey of producing is mentioned but the disadvantages of bioremediation can be mentioned as following:

- The inability of microorganisms to break down all pollutants
- Biodegradation process is slower in compared to other methods
- And producing the toxic metabolites in some cases [8,9].

Microbiological degradation, bioaccumulation and transformation are the main methods for the remediation of the phenanthrene from the environment [10,11]. Several groups of earthworms, bacteria and fungi are able to partly decompose, co-metabolically oxidize or mineralize the phenanthrene to harmless products [12,13]. Earthworms are generally resistant to many chemicals, including heavy metals and organic pollutants in soil [14]. They are usually able to accumulate the phenanthrene in the body wall by intestinal uptake during the passage of soil via the gut [15]. Several earthworm species have been found to remove heavy metals, pesticides and lipophilic organic micropollutants like the phenanthrene from the soil such as *Eisenia fetida*, *Eisenia Andrei*, *Eiseniella tetraedra, Pontoscolx corethrurus, Lumbricus terrestris, Lumbricus rubellus, Dendrobaena rubida, Dendrobaena veneta, Aporrectodea tuberculata* and *Allobophora chlorotica* [16,17]. Earthworms stimulate and increase microbial activity by creating the favorable conditions for bacteria and improving soil aeration. This can be used to remedy the contaminated sites and can occur on- or off-site, which is facilitation by mixed microbial consortia and/or pure microbial strains and plants [18]. These organisms have an important influence on the distribution and activities of the soil micro flora, also they modify the structures, physical and chemical features of soil and act in the organic matter (O.M) degradation, nutrient cycling, chemical exchange and humidity holding through ingesting, burrowing, and casting activities [16]. During the Vermiremediation process of the soil, the population of earthworms increases for benefiting significantly the soil remediation in several ways included promoting the fragmentation, soil aeration and soil turning and scattering brings. Earthworms accumulate many Polycyclic Aromatic Hydrocarbons (PAHs) from the soil, not only throughout inactive absorption of the dissolved fraction through the body wall, but also by intestinal uptake during the passage of soil through the gut [19]. Castellanos et al. (2013) showed that the earthworm (*Pontoscolex corethrurus*) can be used in bioremediation of hydrocarbon contaminated soil [4]. Monard et al. (2008) found that earthworms (*Lumbericus terrestris*) could improve atrazine degradation by changing the microbial community structure of indigenous microorganisms and species bioaugmented [20]. Moreover, Butenschoen et al. (2009) showed that earthworms improved the microbial activity and thus mineralization of phenolic compounds [21].

There are few bacterial strains with the capacity of the degradation of phenanthrene [22-24]. Bacteria are capable of degrading hydrocarbons, but when the earthworms are located in the soil, they will improve the ventilation, motivate the microbial action and therefore biodegradation may be enhanced [25,26]. Previous study indicated that, the bioaugmentation was as an effective method to enhance the bioremediation in removal of phenanthrene from contaminated soils [27,28], but Yu et al. (2005) stated that there was no significant difference between natural attenuation and bioaugmentation in phenanthrene degradation due to the negative interaction between the inoculums and the indigenous microflora [29].

The objectives of this study were: 1) To determine the phenanthrene removal from contaminated soil by co-existing of earthworm and bacteria; 2) to explore the effect of OM content on the phenanthrene removal efficiency by earthworms and bacteria.

## Material and methods

### Chemicals

Phenanthrene and acetone were obtained from Aldrich chemical company (USA), with a >98% purity and J. T. Baker (USA) with purity 99.7%, respectively. Solvents used for extractions include the acetone and methanol (Merck, Germany) which were of high performance liquid chromatography (HPLC) grade, and other chemicals were of analytical grade.

### Experimental set up

In this study, the soil samples were obtained from ranges (0–20 cm depth) of Babol city, province of Mazandaran, Iran. Sampling procedure was previously described by Lee et al. [30]. The chemical and textural characteristics of the soil samples were analyzed in Soil laboratory (Table 1). It was air-dried and sieved with a pore size of 2 mm. The samples were then spiked with dissolved phenanthrene in aceton at concentrations in the ratio of 10 and 30 mg/kg of dry soil. To make sure that the pollutant was equally distributed in the soil samples, it was completely blended. The pots were stored at room temperature (25°C) for five days to ensure that the whole acetone was evaporated. The amount of 400 gr of the spiked soil was put into the each plastic pot with a height of 15 cm and a diameter of 12 cm. Then, bacteria and earthworms added in the pots according to the concentration (10^7^ and 10^8^ numbers per gram soil) and number (10, 20 numbers per pot), respectively. The *Staphylococcus epidermidis*ATCC_14990_ and *Bacillus subtilis*ATCC_6051_ (Institute Pastor, Iran) were cultured in the Brain Heart Infusion (BHI) broth (Merck, Germany), and incubated in the 24 hours at 37°C. McFarland standard was used as a reference to the microbial injection adjusts the turbidity of bacterial. Mature earthworms (*Eisenia fetida*) were manually collected from the soil and cow manure in the north of Iran. Adult earthworms (each with a fresh weight of 0.5 ± 0.1 g) were selected for experiments and the worms were located on a moist filter paper in the dark for 24 hours to avoid their gut content and rinsed with water before the use of them. Earthworms were placed in the two series (ten and twenty numbers) per pot with soil.

**Table 1:** **The chemical and texture characteristics of the soil**

**Analysis**		**Results**
Chemical	CEC	17.4 (cmol/kg)
	pH	7.16
	O.M	5.4%
	O.C	3.15%
	TN	0.25%
	P	0.032%
Texture	Sand	24%
	Silt	60%
	Clay	16%

**CEC** = cation exchange capacity, O.M = organic matter, O.C = organic carbon, TN = total nitrogen, P = phosphorous.

For each concentration modes of phenanthrene, four approaches including natural, only bacteria, only *Eisenia fetida* and bacteria + *Eisenia fetida* were used that three levels of organic matter (2, 5 and 8%) were added to each treatment*.* These approaches were listed in the Table 2. In the present study, 160 pots were used and each of the mixtures was repeated triplicate. Experimental design for 36 treatments was set up using the Design Expert (version 7.0) in the full factorial model. The samples were analyzed after 8 weeks.

**Table 2:** **Treatment of experimental design**

**State if condition**	**T0**	**T1**	**T2**	**T3**
Natural mode	+			
Bacteria (staphylococcus epidermidis + bacillus subtellis)		+		+
Earthworm (Eisenia fetida)			+	+

### Phenanthrene analysis

For extraction of phenanthrene from the soil, method of 3550B EPA was used with some modification that was previously defined by Sheng-wang et al. (2008) [31]. Soil samples were carefully collected and homogenized. 2 grams of samples were placed in an Erlenmeyer flask and mixed with 10 ml acetone. Subsequently, 2-minute ultrasonic (cleaning, UK) of these samples and magnetic mixture (Hidolf, Germany) were done during1 hour at a velocity of 200 rpm. The sample was purified with the micro filters with a pore size of 0.22 μm. The chemical analysis was performed using the High Performance Liquid Chromatography (HPLC, CECIL 4100, USA) tool having a C18 column with a length of 25 cm, an internal diameter of 4.2 mm, a UV/VIS detector and a mobile phase of methanol, water v/v 80:20), operating at a flow rate of 1 ml/min at a wavelength of 220 nm.

### Bacterial counts

Bacterial counts were done by using the serial dilution method. Initially, 1gr of the soil sample was suspended with 10 ml and sterilized in normal saline to access a dilution of 10^−1^. Then, 1 ml of this suspension was injected with 9 ml of normal saline. The serial dilution was done up to 10^−12^. 1 ml of all dilutions was cultivated on nutrient agar (Merck, Germany) by use of pour plate method. The plates were incubated at 35°C for 24 hours and then the colony count was performed and reported as the number of colony-forming units per g dry soil (CFU/g) [32].

### Earthworm’s analysis

For extraction of phenanthrene from the earthworm, the recommendation set by the Organization for Economic Cooperation and Development (OECD, 1984) was used with some modification that was previously defined by Kelsey et al. [33]. To assess the ability of the phenanthrene removal from the soil, the *Eisenia fetida* were used through bioaccumulation after separation of the other earthworms from the soil. Initially, the earthworms were washed by using the tap water, then, weighted by using the digital scaling next, placed in the moist filter paper for 24 hours due to the exertion of the gut. After rewashing, killing of earthworms was done by cooling in the refrigerator and then their corpses transferred into the oven at 65°C for 10 minutes. Then 10 ml acetone was added and mixed for 1 hour in the 200 rpm. After that, the purification and chemical analysis of these samples was performed similar to the phenanthrene analysis in soils. The accumulation of phenanthrene was reported by means of the nanogram per gram of body weight of the earthworm.

### Statistical analysis

After collecting data by the use of Excel 2013 and SPSS (version 14.0), the statistical analysis was performed using the design expert (version 7.0) and One-way Anova test.

## Results and discussion

**Figure 1:**
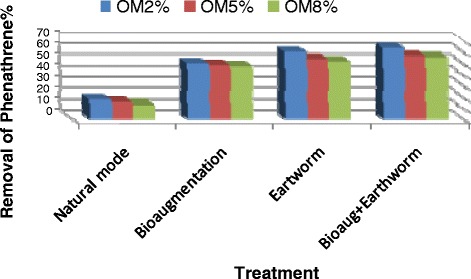
**Percentage of degradation for phenanthrene in different treatment.**

**Figure 2:**
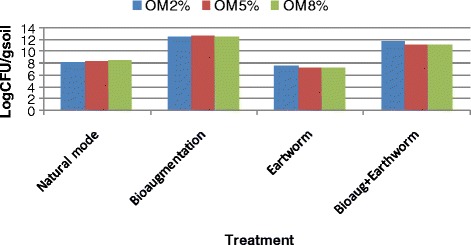
**Bacterial population in different treatment.**

The bioaugmentation and vermiremediation in four treatments were considered for phenanthrene biodegradation (Table 2). Figure 1 demonstrates the mean removal efficiency of phenanthrene in all of the treatments. In free-treatment of earthworms and bacteria (natural mode), the phenanthrene concentration in the soils reduced over the time. Before the decrease of phenanthrene through the biodegradation, its concentration was reduced through other ways such as evaporation and adsorption onto the soil wall [34]. Decrease rate of phenanthrene in natural mode in the presence of 2, 5 and 8% of the O.M was 18.44, 16.5 and 14.18%, respectively. This removal was related to the increased microbial population of the soil by the suitable level of humidity through watering. Soil humidity increased the soil microflora activity and the degradation of contaminants, too [4,17]. The phenanthrene removal by the bacterial consortium predominantly *Bacillus subtilis* and *Staphylococcus epidermidis* (bioaugmentation mode) showed that, the removal of phenanthrene was greater than the natural mode (P value < 0.0001) (Table 3). The results of this study showed that the degradation efficiency in the presence of 2, 5 and 8% of the concentration the O.M was 50.3, 48.86 and 47.64%, respectively. The phenanthrene elimination by the *Eisenia fetida* (earthworm mode) displayed that, the removal of phenanthrene was greater than both natural (P value ≤ 0.0001) and bioaugmentation (P value ≤ 0.035) modes (Table 3). These results showed that the removal efficiency in the presence of 2, 5 and 8 Percent of the concentration of O.M were 60.24, 54.3 and 51.86%, respectively. The combination of the *Eisenia fetida* with the bacteria (bioaugmentation + earthworm mode) showed that this mode was most effective than the others, which is in agreement with the study of Sun et al. [26]. Their results showed that, the Pyrene removal in the presence of *E. fetida* was 2.1 to 2.8 times greater than those without the worms. They attributed it to both enhanced microbial degradation and uptake by the worms. They were indicated Microbial degradation of Pyrene increased by 1.2 to 1.6 times in the presence of the worm [26]. The results of statistical analysis showed that the presence of earthworms significantly (P value ≤ 0.0001) increased the phenanthrene removal from soil (Table 3). The removal rate in the presence of 2, 5 and 8% of O.M was 63.81, 56.86 and 55.5%, respectively. Therefore, in the presence of earthworms, biodegradation increased and the effect of earthworm improvement in the remediation proficiency of phenanthrene could be attributed to not only the accumulation of phenanthrene in earthworm bodies but also the strengthened biodegradation by microflora. The findings indicated that the rate of biodegradation of organic pollutants in the presence of earthworms was more than natural and bioaugmentation modes. These results are consistent with the studies of Hickman et al. and Butenschoen et al. [21,35] and support the fact that the phenanthrene-degrading bacteria and earthworms can effectively cooperate to remove the phenanthrene from soils by the worm uptake and enhance the biodegradation because of earthworms' stimulation and better microbial activity by creating favorable conditions for bacteria and soil aeration [18]. Statistical analysis showed that the natural mode and combination of the bacteria and earthworm mode had the lowest and highest efficiency on phenanthrene removal, respectively (p ≤ 0.0001) (Table 4). The bacterial count in natural mode was in the range of 10^4^-10^6^ CFU/g in the soils and this count in different treatment is shown in the Figure 2. Degradation in the presence of high concentration-organic-matter level in soil was lower than the lowest concentration of O.M content. This was according to the study of Greer et al. (1994) that indicated the rates of degradation in the high-organic-matter level in soil were lower than the low-organic matter soil, therefore, there was a negative correlation between OM contents and the extracted fraction of phenanthrene [36]. The colony count in the bioaugmentation style was in the range of 10^9^-10^12^ CFU/g in the soils. This finding was in agreement with that of Baneshi et al. and Hunter et al. [28,37], they said that the bioaugmentation by this bacteria improved the phenanthrene degradation because of the high number and their ability in petrol biodegradation compared with the natural mode. The colony count of the earthworm mode was in the range of 10^4^-10^6^ CFU/g in the samples. Due to the existence of the earthworm in the samples, the numbers of bacteria were reduced owing to the anti-microbial peptides (AMPs) production by the earthworm. AMPs were released naturally in response under injury or stress. The results were in accordance with those of Bhorgin et al. (2014) and Laverty et al. (2011) [38,39]. The bacterial count in the bioaugmentation and vermiremediation mode was in the range of 10^7^-10^12^ CFU/g in the soils. Microbial activity in the presence of earthworms was improved and it might be due to the soil digging by the worms, which was more desirable for bacterial growth and improved ventilation or because the bacteria were more distributed, they can better degrade the phenanthrene. During this study, it was perceived that the texture of the earthworm mode soil gradually differentiated into a more homogeneous and finer one and the soil humidity stayed constant while the worm in free mode soil protected and its original structure condensed and its moisture content decreased quickly. Previously, similar studies were an agreement with the current study [40-42] because the soil humidity increased the microflora activity of the soil and hence degradation of contaminants [22,28]. In this study, phenanthrene removal decreased from the soil by increasing of the O.M content. It seems that the bacteria and earthworms tend to use the organic matter as a nutrient than the phenanthrene, therefore, the phenanthrene remains in the environment and less degrades.

**Table 3:** **Post Hoc Test for phenanthrene removal efficiency**

**(I) Treatmen**	**(J) Treatment**	**Mean ± SD (removal percentage)**	**Std. error**	**P-value**
Natural Mode	Bacteria	48.93 ± 1.78	3.69536	≤0.0001
	Earthworm	55.47 ± 9.11	3.69536	≤0.0001
	Bacteria + earthworm	58.73 ± 8.72	3.37338	≤0.0001
Bacteria	Natural Mode	16.37 ± 3.69	3.69536	≤0.0001
	Earthworm	55.47 ± 9.11	3.01724	0.035
	Bacteria + earthworm	58.73 ± 8.72	2.61301	≤0.0001
Earthworm	Natural Mode	16.37 ± 3.69	3.69536	≤0.0001
	Bacteria	48.93 ± 1.78	3.01724	0.035
	Bacteria + earthworm	58.73 ± 8.72	2.61301	0.218
Bacteria + earthworm	Natural Mode	16.37 ± 3.69	3.37338	≤0.0001
	Bacteria	48.93 ± 1.78	2.61301	≤0.0001
	Earthworm	55.47 ± 9.11	2.61301	0.218

**Table 4:** **Anova for phenanthrene removal efficiency**

	**Sum of squares**	**df**	**Mean square**	**F**	**Sig.**
Between groups	8917.932	3	2972.644	54.422	<0.0001
Within groups	2731.130	50	54.623		
Total	11649.062	53

## Conclusions

The results of the current study indicated that the bacteria and earthworms could effectively cooperate to remove phenanthrene from soils in two approaches: earthworm uptake and enhanced biodegradation. More experiments about the mechanisms of the actuated biodegradation showed that earthworms led to enhance the microbial activity and an increased bioavailability of phenanthrene, that in turn worked synergistically of facilitate the microbial degradation of phenanthrene in soils. The current study supports the use of earthworms, together with microbial degradation technologies, to produce the comprehensive, innovative remediation approach, according to ecological roles in the removal of carbon-based pollutants.
